# Specim IQ: Evaluation of a New, Miniaturized Handheld Hyperspectral Camera and Its Application for Plant Phenotyping and Disease Detection

**DOI:** 10.3390/s18020441

**Published:** 2018-02-02

**Authors:** Jan Behmann, Kelvin Acebron, Dzhaner Emin, Simon Bennertz, Shizue Matsubara, Stefan Thomas, David Bohnenkamp, Matheus T. Kuska, Jouni Jussila, Harri Salo, Anne-Katrin Mahlein, Uwe Rascher

**Affiliations:** 1 INRES-Plant Diseases and Plant Protection, University of Bonn, 53115 Bonn, Germany; jbehmann@uni-bonn.de (J.B.); stefan.thomas@uni-bonn.de (S.T.); davidb@uni-bonn.de (D.B.); mkuska@uni-bonn.de (M.T.K.); amahlein@uni-bonn.de (A.-K.M.); 2 IBG-2, Forschungszentrum Jülich (FZJ), Jülich, 52428 Germany; k.acebron@fz-juelich.de (K.A.); d.emin@fz-juelich.de (D.E.); s.bennertz@gmail.com (S.B.); s.matsubara@fz-juelich.de (S.M.); u.rascher@fz-juelich.de (U.R.); 3 Institute of Sugar Beet Research (IFZ), 37079 Göttingen, Germany; 4 Specim Ltd., FI-90571 Oulu, Finland; jouni.jussila@specim.fi (J.J.); harri.salo@specim.fi (H.S.)

**Keywords:** hyperspectral camera, handheld, sensor evaluation, case studies

## Abstract

Hyperspectral imaging sensors are promising tools for monitoring crop plants or vegetation in different environments. Information on physiology, architecture or biochemistry of plants can be assessed non-invasively and on different scales. For instance, hyperspectral sensors are implemented for stress detection in plant phenotyping processes or in precision agriculture. Up to date, a variety of non-imaging and imaging hyperspectral sensors is available. The measuring process and the handling of most of these sensors is rather complex. Thus, during the last years the demand for sensors with easy user operability arose. The present study introduces the novel hyperspectral camera Specim IQ from Specim (Oulu, Finland). The Specim IQ is a handheld push broom system with integrated operating system and controls. Basic data handling and data analysis processes, such as pre-processing and classification routines are implemented within the camera software. This study provides an introduction into the measurement pipeline of the Specim IQ as well as a radiometric performance comparison with a well-established hyperspectral imager. Case studies for the detection of powdery mildew on barley at the canopy scale and the spectral characterization of Arabidopsis thaliana mutants grown under stressed and non-stressed conditions are presented.

## 1. Introduction

**Figure 1:**
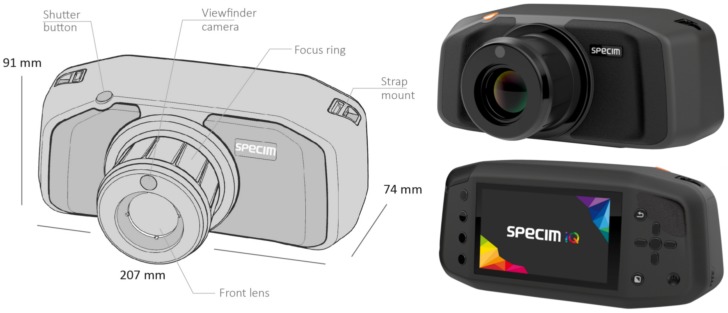
Vector visualization of the Specim IQ (Specim Ltd., Oulu, Finland) with annotations and dimensions on the left side and RGB renderings on the right side.

This study introduces the novel, handheld hyperspectral camera Specim IQ and evaluates it with respect to applications in plant physiology and plant pathology. The sensor is designed as a mobile, hand-held and stand-alone camera (cf., Figure 1) with ground-based application areas. For monitoring the status of plants, field, greenhouse and laboratory-based measurements can be performed. By measuring spectral reflectance from 400 to 1000 nm, several important plant traits can be covered by the sensor. The sensor relies on the established push broom principle but integrates a scanner system and allows focusing in the image plane. All investigations presented in this publication were performed on a prototype of the Specim IQ sensor and by using the corresponding software Specim IQ Studio during the pilot phase.

Hyperspectral imaging (HSI) is a non-invasive method, which can provide detailed and highly resolved reflectance characteristics of target materials on different scales. The reflected light of the target is recorded with a high spectral and spatial resolution of a two-dimensional image [1]. In hyperspectral images, every pixel has more than hundred consecutive bands, containing the reflectance values of the target for specific wavelengths called the spectral signature [1]. Many sensors address the visible part of the electromagnetic spectrum (400–700 nm, VIS) and are also able to measure the near-infrared wavelength (700–1000 nm, NIR) specifying them as VISNIR sensors. The shortwave-infrared (1000–2500 nm, SWIR) part of the electromagnetic spectrum is also important for specific applications but typically not all spectral regions can be covered by one sensor.

In recent times, an increasing number of successful studies prove the high versatility of applications of hyperspectral imaging. Differences in the material composition of the measured target—e.g., living plants, fruits, geological samples—are reflected in the corresponding spectral profiles. This allows the detection of important parameters in food production, soil science and precision agriculture [2,3,4,5,6]. Hyperspectral imaging is applied for an improved quality and safety monitoring in food production [7,8,9]. Successful applications have been shown e.g., for lesion detection on apple skin using a hyperspectral NIR camera [10], prediction of moisture in soybeans by VISNIR imaging [11] and the acidity levels of grapevines from seven varieties by NIR hyperspectral imaging [12]. Pigment concentration and other quality parameters of bell pepper were determined using VISNIR imaging and Partial Least Squares Regression [13]. In soil science, hyperspectral imaging is used for the investigation of soil composition and evaluation of soil quality. The spatial distribution of three classes of organic matter within 10×30 cm soil samples was determined using k-means clustering [3]. In a similar approach, a snapshot camera with the potential for outdoor application was applied for visualizing the spatial heterogeneity and estimating soil properties like nitrogen or clay content by Partial Least Squares Regression [2]. Using the SWIR range, a spectral library has been developed that allows the detection of crude oil contamination in different soil types [14].

In precision agriculture, HSI can be applied for site-specific fertilization and precise plant protection applications [15,16,17]. In recent years, HSI was introduced in greenhouse and field phenotyping for non-invasive quantification of structural and functional plant traits [18]. Integrated multi sensor platforms allow the application in the field, e.g., for grapevine phenotyping [19]. Studies in plant pathology showed the advantage of a high spatial resolution in close range imaging to detect and characterize plant diseases and even subtle resistance mechanisms of plants to diseases [20,21]. On the other side of the scale, hyperspectral sensors are increasingly used for ecosystem monitoring and remote sensing of vegetation [22,23]. In this context, Vegetation Indices (VIs) are commonly used to remotely evaluate vegetation covers both quantitatively and qualitatively [24,25]. These spectral ratios were shown to be sensitive to changes in plant functional status which helps in estimating gross [26,27] and net photosynthesis [28] down to its efficiency [29].

With the increase of different application scenarios, the demand for tailored hyperspectral cameras rises. Every hyperspectral sensor type has specific strengths and limitations, which need to match the requirements of the measured object and aim of investigation. Push broom and whisk broom scanners capture the spectral information of a line or point on the measured object, respectively. To compose the hyperspectral image, the object is scanned through movement or rotation [21]. However, the required scanner process limits the application when the object is in motion.

Airborne applications mostly aim at plant status sensing or variety mapping and rely on whisk or push broom systems. The spatial referencing is performed with suitable accuracy by using additional correction parameters from an inertial measurement unit and global navigation satellite systems [30]. Light-weight platforms, like UAVs, have the advantage of lower flight altitudes but do not provide such high-quality correction signals.

Triggered by the trend towards UAVs, full frame and snapshot hyperspectral cameras arouse intense attention. Usually, the image data is spatially referenced by established structure-from-motion software in post processing [31]. The underlying measurement principles are well-known but now significant technical development allows to produce light-weight, reliable sensors with sufficient radiometric accuracy for many applications. The main approaches for multi- and hyperspectral frame cameras are spectral scanners [32], multi-point spectrometer [2], mosaic sensors and multi-sensor systems. The Rikola hyperspectral camera (Senop, Kangasala, Finland) as a Fabry-Pérot-filter camera specifies the wavelength of the light illuminating the panchromatic sensor array [32]. By fast changing of the passing wavelength, hyperspectral images of up to 1010×1010 px and 380 bands can be captured in the current version of the camera. A particular feature of the camera is to define the needed bands and to reduce measurement time by reducing the number of bands. Multi-point spectrometers, such as the Cubert S 185 (Cubert GmbH, Ulm, Germany), are capturing the whole spectral characteristics in one shot by projecting the spectra of different pixel locations onto one 2D sensor [32,33]. The low spatial resolution of the hyperspectral image, 50×50 px and 125 bands for the Cubert S 185, is partially compensated by an additional panchromatic image with higher spatial resolution allowing pan-sharpening. Mosaic cameras in contrast are basically multispectral cameras that expand the principle of common RGB cameras by adding more and narrow band filters in front of individual pixels [34]. In one image frame, up to 25 bands can be captured. A variant uses a line layout capturing up to 70 bands in one shot but only one band for each pixel location [35]. To restore a hyperspectral image, proper referencing and 3D reconstruction with multiple images at different positions are necessary. Other multispectral designs integrate up to six sensor arrays, each sensing up to three bands. They can be classified as multi camera systems with several optics and sensor arrays (e.g., the popular TetraCam [36]). The Specim IQ shares some characteristics with these snapshot cameras especially with the spectral scanner, e.g., capturing a full hyperspectral image without external movement and the slight temporal delay of capturing different parts (spatial or spectral) of the image. Latter will cause distortions if a moving object is observed. The Specim IQ may allow UAV applications as well, but due to its focus on direct manual control it is not possible in the current version.

Ground-based hyperspectral sensing includes applications in the field, the greenhouse and the laboratory with moving or static platforms. Moving field platforms and field robots rely mainly on line scanner systems as the camera weight is a minor factor and the movement is already provided by the platform [17,37,38]. Distortions as a result of vibrations do not affect the spectral signal, but only affect the spatial image and are typically not corrected. For chlorophyll estimation in sugar beet for example, a moving platform was combined with a linear stage [39]. A similar set-up was also recently adopted to use high performance and thus heavy imaging spectrometers to retrieve subtle differences between different breeding lines in the field [40,41]. In the greenhouse, more compact measurement stations are required. For water stress detection, a line scanner camera was combined with a rotating mirror to obtain hyperspectral images [42]. In Reddy et al. [43], a line scanner was used with a linear stage in the greenhouse and furthermore, the same line scanner was attached to a tractor for field observations in order to detect glyphosate-resistant weeds.

In the laboratory and in high-throughput systems, the use of line scanners is common as under optimal measurement conditions the spatial and spectral measurement quality is superior [21,39,44]. Ge et al. [44] derived a variety of plant-physiological parameters using a conveyor belt-based high-throughput facility with a hyperspectral sensor cabin. A mirror-based push broom sensor was used to capture images of single sugar beet leaves and to derive disease specific vegetation indices [21]. Jay et al. [39] used a fixed camera with attached illumination while the plant probe was moved by a translation stage. They obtained multi-angle data and derived radiative transfer models especially suited for the close range. Microscopic observations for the investigation of resistance reactions of a plant against a fungal pathogen were also observed by a line scanner attached to a microscope optic [20]. The image was gathered by moving the probe during the measurement.

High amounts of spectral data are collected with each hyperspectral image, making it difficult to extract relevant information from the images. This leads to the requirement of advanced data analysis methods in order to work efficiently with hyperspectral sensors [45]. For a successful application, a suitable sensor needs to be accompanied by an appropriate data analysis pipeline, which needs to be concerted to the sensor characteristics.

In the present study, the characteristics of the novel hyperspectral camera Specim IQ are presented, and a direct qualitative comparison on radiometric accuracy with the well-established sensor Specim HS-V10E-CL-30 [21,40,41,46,47] (denoted in the manuscript as Specim V10E (Specim Ltd., Oulu, Finland)) was conducted. Further details on this sensor are provided in Section 3. The IQ sensor is accompanied by the software Specim IQ Studio (Specim Ltd., Oulu, Finland) which relies on the Spectral Angle Mapper (SAM) algorithm to analyze the images and to develop SAM applications which can be executed on the device itself. The performance of the sensor and the accompanying data analysis software were investigated in two lifelike case studies. Performance for the detection of powdery mildew on barley and classical differentiation of *Arabidopsis thaliana* mutants are demonstrated.

## 2. Technical Description of the Specim IQ Camera System

This section provides the technical background of the sensor and gives an overview of the different normalization possibilities and measurement modes. Specim IQ is a handheld hyperspectral camera, which performs hyperspectral data capturing, data processing and visualization of a classification result in one single integrated unit. The camera is supported by Specim IQ Studio software . Using the software, the user is enabled to develop and download its own applications to the camera as it possesses embedded processing capability. Detailed technical specifications are given in Table 1.

The measurements are performed based on the line scanner, i.e., push broom principle and comprise the wavelength range 400–1000 nm. Its spatial sampling, i.e., the number of pixel per line is 512, and the spectral resolution is 7 nm with 204 spectral bands across the wavelength range. By adjusting the binning in spectral dimension, the amount of spectral bands can be adjusted. The number of imaged lines is static with 512 lines and thus the camera captures always a square image with a resolution of 512×512 px. Specim IQ camera is equipped with a touch display, physical buttons, chargeable battery, replaceable memory card and USB connector. The camera employs a processor (NVIDIA Tegra K1 (NVIDIA Corporation, Santa Clara, USA)), spectral camera (CMOS technology), viewfinder camera (5 Mpix), focus camera and a scanner with the motor for optics movement. The fore optic of the camera provides a 31${}^{\circ }$× 31${}^{\circ }$ field of view and focusing is set manually. The focus range of the camera is from 150 mm to infinity. As an example, with 1 m distance to the target, the viewable area is 0.55 × 0.55 m, resulting in a spatial resolution of 1.07 mm on the target.

**Figure 2:**
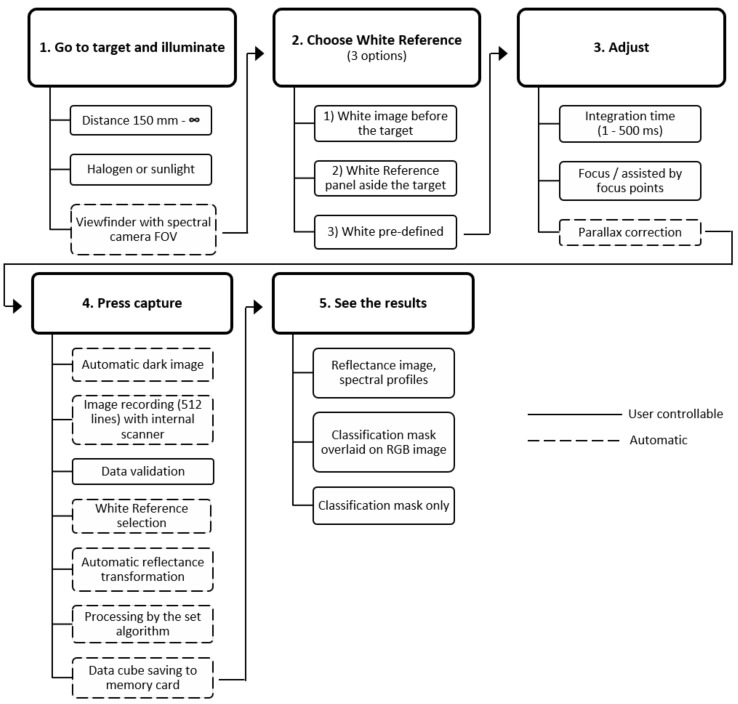
The standard workflow of the Specim IQ hyperspectral camera.

Specim IQ is controlled via a touch screen and relies on a graphical user interface that guides the user through the entire imaging pipeline (Figure 2). The usability has been designed to have similarities with a standard system camera and to require only minimal user input. One main character of the camera is its ability to convert the recorded hyperspectral data into instant classification results displayed on the screen. For this purpose, the Specim IQ uses a workflow which is shown in Figure 2.

The measurement process is divided into five steps. During the establishment of the imaging setup, the hyperspectral camera is directed to the target using the viewfinder camera, i.e., a RGB camera with identical viewing direction and small vertical offset. As common for any hyperspectral measurement, a suitable illumination providing a continuous spectrum over the wavelength range of interest is required. This could be sunlight outdoors or halogen lamps indoors. Typically, the characteristics of the illumination are captured by the measurement of a white reference panel, which has to be located next to the sample.

After this, the integration time and camera focus needs to be adjusted. The viewfinder camera image gives a preset for the integration time of the spectral camera. The preset value and a preferable range are proposed to the user, but a manual adjustment of the integration time in the range 1–500 ms is possible. The focusing of the system is done manually, but supported by the focus camera image. In contrast to a traditional line scanner camera, this approach uses a normal camera image and highlights sharp edges. The parallax between the viewfinder camera and actual spectral camera is compensated by an automatic calibration done by edge detection between viewfinder and focus cameras. The vertical position of the viewfinder camera image is virtually adjusted accordingly or alternatively, based on a manual parallax correction. The adjustment is needed to overlay the spectral and viewfinder camera images with suitable accuracy.

After these initial adjustment steps, image recording is started by pressing the capture button. At first, the dark reference, representing the sensor noise without incoming light is recorded automatically. This is done on the spectral camera home position that is blocked from incoming light. After the dark reference acquisition, the spectral camera is moved to the measurement starting position and the actual data acquisition is started.

After the full 512 × 512 px image is scanned, the user can check focus and integration time in the data validation view. There, a RGB image derived from the hyperspectral image is shown together with an intensity histogram. Based on the validation view, the image can be rejected, kept for the further workflow or it can be selected to save only raw, uncorrected data. For the white reference measurement, Specim IQ offers three possibilities. The white reference can be recorded prior to the actual measurement (custom white reference). Alternatively, the white reference can be recorded simultaneously with the actual sample (simultaneous white reference). In cases where white reference recording is not possible, device memory provides a general halogen spectrum from 400 to 1000 nm (pre-defined white reference). In the first two possibilities, an area scan over the white reference is performed, in which 100 random spectra are averaged and used for data reflectance transformation. The latter approach uses the pre-defined spectrum directly to convert the measured data to reflectance. In all cases, the reflectance transformation is performed automatically for the data.

For data processing, Specim IQ provides three different modes: *Default Recording Mode* (without any processing), *Automatic Screening Mode* (ASM, one-class classifier) and *Application Mode* (deployed processing model developed by Specim IQ Studio).

In *Default Recording Mode*, all the raw data and reflectance data are saved into separate data cubes on the data set. The data set is a folder structure, where the data is stored together with meta information. The ASM can be used to build a single class classifier, at the site, and directly from the user interface of the Specim IQ. The ASM mode is based on a Spectral Angle Mapper [48]. The user has a possibility to select the reference spectra, as well as threshold for class creation. Prior to the classification, the reflectance transformation is performed as described above, as well as automatic wavelength range selection to avoid using noisy parts of the spectra. By using the Specim IQ Studio software, it is possible to build applications that can be uploaded to the Specim IQ camera. In the *Application Mode* it is possible for the user to define data recording, visualization and saving. Furthermore, the data classification can be defined by creating several classes, class groups, data preprocessing (Savitzky–Golay algorithm for data smoothing [49]), and classification for the targeted application. The Specim IQ Studio software allows to create applications without any programming directly from a graphical user interface.

If Specim IQ is used in the *Default Recording Mode*, the RGB image derived from reflectance data is shown to the user after measurement. There are possibilities for the user to check the spectra, add tags and other information to the dataset. If the data processing capabilities of Specim IQ are used, either with *Automatic Screening* or *Application Mode*, the classification mask is visualized as an overlay on a RGB image or without RGB information. Using this visual feedback, a direct assessment of imaging quality and flexible real-time analysis of samples using hyperspectral images is possible. Importing own spectral algorithms beyond the SAM to Specim IQ is not possible and models in Specim IQ Studio are limited to SAM only. However, importing reference spectra for the basis of SAM classification model is supported and processing time can be optimized with wavelength range selection and averaging spectral bands.

## 3. Qualitative Comparison of Specim IQ with Specim V10E

Comparative measurements of the Specim IQ system and Specim V10E sensor were conducted to assess the measurement quality of the Specim IQ camera and to provide an impression for which applications the new sensor is suited.

The Specim V10E sensor is an established and representative system of the class of hyperspectral push broom cameras in the same spectral range (400–1000 nm) as the Specim IQ. In this study, four-fold spectral binning is applied to provide a similar sampling distance using 211 bands. Capturing 1600 pixel per line, it has in general a higher spatial resolution than the Specim IQ, but it is dependent on an external movement which is provided by a linear stage in the present setup [21,47]. The sensors were compared in an indoor setting with halogen illumination and an outdoor setting with natural light to capture the measuring performance for multiple relevant scenarios. Both sensors were positioned in nadir orientation above different test materials, such as paper and polyethylene of different color (in the following called color cards). In the laboratory, the Specim V10E—including the light sources—was moved by a linear drive across the object of interest whereas the Specim IQ used the internal scanning properties and a spatially fixed illumination.

During outdoor measurements, sunlight from full-midday sun (~90.000 lx) was used as the only light source. In both scenarios, the cameras were placed as close to each other as possible with a maximum distance of 20 cm during the movement of the line scanner. The test materials were placed at a distance of 1 m. All images were normalized using a 99% barium sulfate white reference (Specim), which was positioned within the field of view during image acquisition. Due to the different measurement principles (push broom vs. static), the comparability of measurement conditions is limited by the spatially variable light conditions in the laboratory. As a result of the moving light sources of the Specim V10E, both cameras observed the white reference with slightly different light intensity. To remove this effect and obtain comparable reflectance values, the spectral signatures of the Specim IQ were scaled to attain the same mean reflectance value as the Specim V10E spectra.

**Figure 3:**
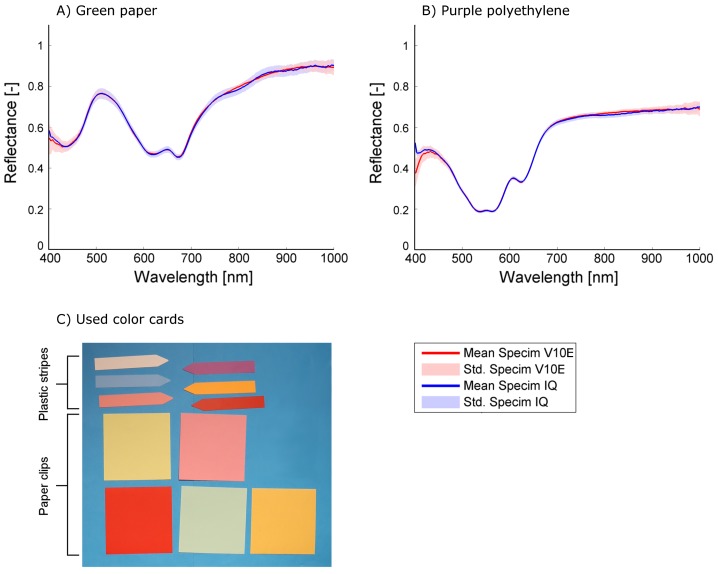
The mean spectra including the standard deviation of green paper (**A**) and purple polyethylene (**B**) as representatives of the observed reference objects of different color in the indoor setting (**C**).

**Figure 4:**
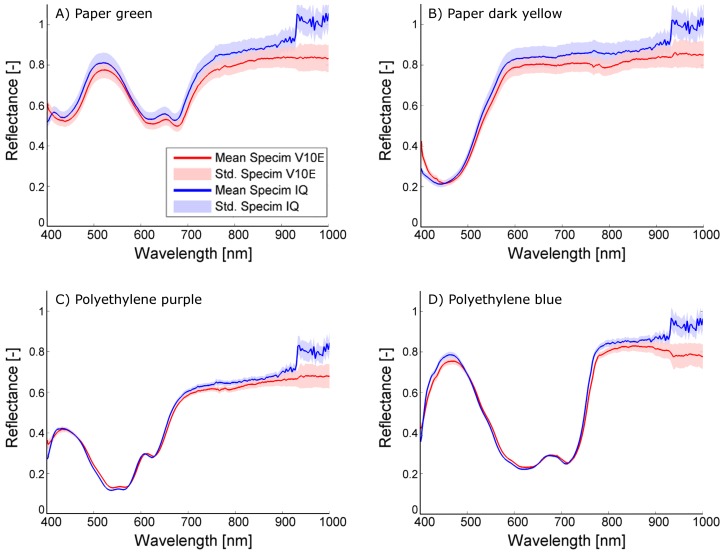
Comparison of spectral reflectance spectra from the outdoor experiment (**A**–**D**): paper green, paper dark yellow, Polyethylene purple, Polyethylene blue. Beside the increased reflectance observed by the Specim IQ in the NIR, all spectra reveal a high level of congruence.

The mean reflectance, its standard deviation and the mean and maximum distance between the Specim IQ reflectance spectrum and the Specim V10E reflectance spectrum for all color cards were calculated from more than 400 px. A subset of representative spectra comparing the sensor performance is given in Figure 3 for indoor measurements and in Figure 4 for outdoor measurements. All parameters illustrating the accuracy and the concordance of both sensors using all color cards for the laboratory and for the outdoor scenario are given in the appendix.

The results allow to group the materials in paper and polyethylene which show a low in-group variability of the accuracy parameters (Table A1). In the indoor experiment, a mean absolute distance between the spectra of Specim V10E and Specim IQ of 0.007 and 0.009 and maximum absolute distance of 0.025 and 0.030 was observed for the paper and polyethylene materials, respectively. The standard deviation of 0.017 for the Specim V10E and 0.021 for the Specim IQ represents the combined effect of measurement noise and material heterogeneity. In the outdoor experiment, a mean absolute distance of 0.054 and 0.032 and maximum absolute distance of 0.0207 and 0.164 was observed for the paper and polyethylene materials, respectively (Table A2). In contrast to the indoor experiment, the standard deviation of 0.038 and 0.020 for the Specim V10E and 0.042 and 0.017 for the Specim IQ reveals significant differences between the two material groups. The increased standard deviation of the paper color cards measured outdoors is presumably caused by angular effects as the paper was not completely flat due to fixation. As the same parts of the paper cards are averaged in the images of both sensors, the majority of this effect is excluded from the further analysis.

**Figure 5:**
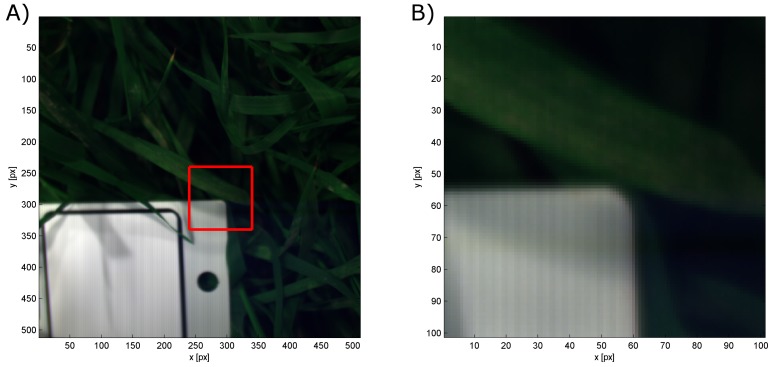
RGB visualization of a reflectance test image to show the line pattern. The highlighted image part in (**A**) is visualized in zoom view in (**B**). On the white reference the line pattern is visible, whereas on the plants it is mainly covered by natural variability.

Overall, it turns out that the sensors give comparable results. The shape of the observed spectra corresponds to a high degree, resulting in mean differences of 0.009 in the laboratory and 0.043 outdoors. The mean standard deviation of Specim IQ and Specim V10E are on the same scale indoors (0.017 and 0.021) and identical outdoors (0.029 and 0.029), but for the Specim IQ it is more homogeneous whereas the Specim V10E has a higher noise level in the spectral border regions (400–450 nm and 900–1000 nm, cf. Figure 3A). As negative characteristics, outdoor observations with the Specim IQ showed an increased reflectance between 925 and 1000 nm. The remaining part of the spectrum was not affected. This results in comparably high maximum deviations (on average 0.186) and may be related to the low integration time of 1 ms. According to the manufacturer, this effect was caused by the low raw signal level in addition to the atmospheric (H_2_O) absorption band in the range between 925 and 970 nm. This results in the prompt increase in the reflectance signal. The remedy for this would be to use spectral flattening filter in front of the fore optics and increase the integration time. Furthermore, a line pattern was visible in all images (Figure 5). According to the manufacturer, it is induced by the changing optical geometry at the slit during the scanning process. As a consequence, the slit width is not completely identical for single lines of the image. Nevertheless, due to the averaging of multiple lines, the mean spectra were not affected.

## 4. Case Studies

In addition to the qualitative evaluation in comparison to an established sensor, the performance of the Specim IQ was investigated in two case studies. In the first case study, the reflectance characteristics of three different genotypes of *Arabidopsis thaliana* were recorded to test the capacity of the camera to resolve subtle differences that are based on slightly different leaf pigment composition. The second case study aimed to quantify the level of infestation of two barley cultivars inoculated with powdery mildew, using the machine learning method Support Vector Machine (SVM [50]). By this selection, two relevant applications in plant and crop science, dealing with different plant functional traits were investigated.

### 4.1. Use of Specim IQ Camera System as a Tool for Understanding Physiological Response of Arabidopsis thaliana Mutants Adapted in Stressed and Non-Stressed Condition

Vegetation indices (VIs) are used by plant scientists to evaluate structural and functional vegetation traits quantitatively and qualitatively across different scales [24,25,51,52]. They are used for example to assess the green biomass [53], canopy structure [54], leaf area index [55], chlorophyll content [56,57,58], fraction of absorbed photosynthetically active radiation (fAPAR) [55,59], and light-use efficiency [29] of plants and canopies [60]. With the development of portable hyperspectral imaging sensors, reflectance from visible (400–700 nm) and near infrared (700–1000 nm) spectrum can be easily derived to study and screen mutant plants to identify genes and understand its physiological function in a high-throughput way in phenotyping environments.

In this case study, two mutant lines of *Arabidopsis thaliana* acclimated in non-stressful (NSA) and stressful (SA) condition were used as the main subject. The selected mutants are deficient in either PsbS protein (*npq4*) [61] or violaxanthin de-epoxidase (*npq1*) [62], both resulting in a limited ability to thermally dissipate excessive light energy via a process called non-photochemical quenching (NPQ). In addition, the lack of violaxanthin de-epoxidase in *npq1* mutant inhibits light-dependent enzymatic conversion of carotenoids. This conversion, which is a part of NPQ regulation, causes a very subtle spectral change in leaf reflectance that is invisible to human eyes. Both mutations are not fatal and the plants develop normally under greenhouse conditions. Under high light conditions, however, the two mutants are unable to adjust light energy harvesting by NPQ to protect their photosystems against photo-damage. Along with these mutants, the “normal” plants without mutations (Col-0) were used as a control group. Thus, by using these mutants we tested whether the Specim IQ can detect subtle differences in leaf pigments and physiological traits.

Plants were sown in 7 × 7 cm pots (one plant per pot) filled with soil. Three plants from each genotype were grown and acclimated to non-stressful conditions in the greenhouse while another three plants were acclimated for at least two days to natural light and temperature conditions in the field that are more stressful for plants. All plants were randomly distributed within the imaging frame.

The Specim IQ camera system was used to take reflectance images of *Arabidopsis* plants inside the greenhouse with white panel (90% reflectance) as a reference target. As the Specim IQ software does not support the calculation of vegetation indices, the hyperspectral imagery data was imported into ENVI Classic 5.3 (Harris Geospatial Solutions, Broomfield, USA) resolving the regions of interest (ROI) using Normalized Difference Vegetation Index (NDVI [63], Equation (1)) and manual tracing of individual plants. For the case study, two VIs were calculated that are correlated to leaf chlorophyll content, namely the NDVI and the Red Edge Inflection Point (REIP [64,65], Equation (2) by Matlab 2013a and Signal Processing Toolbox 6.19 (The MathWorks, Inc., Natick, USA)). To derive REIP, plant spectra was smoothed using Savitzky–Golay filtering [49] before calculating the first derivative. REIP was identified as the maximum value of the first derivative between 690 and 720 nm after spline interpolation with 0.1 nm resolution. As a third VI, the Photochemical Reflectance Index (PRI [66], Equation (3)) was calculated, which is described to be sensitive to the activation of the NPQ pathway and carotenoid conversion (for more details on these indices and their functional meaning see [25]. Using the RStudio software V1.0.143 (RStudio, Inc., Boston, USA), analysis of variance (ANOVA) was carried out using agricolae package for all the calculated average VIs in a single plant. Likewise, pairwise mean comparison using least square difference (LSD, α=0.05) was performed as a post hoc analyses.

(1) $$\begin{array}{cc} \mathrm{NDVI} & =\frac{R_{800}-R_{680}}{R_{800}+R_{680}} \end{array}$$

(2) $$\begin{array}{cc} \mathrm{REIP} & =\underset{690\leq x\leq 720}{\arg\max}\;R_{x}^{\prime } \end{array}$$

(3) $$\begin{array}{cc} \mathrm{PRI} & =\frac{R_{531}-R_{570}}{R_{531}+R_{570}} \end{array}$$

**Figure 6:**
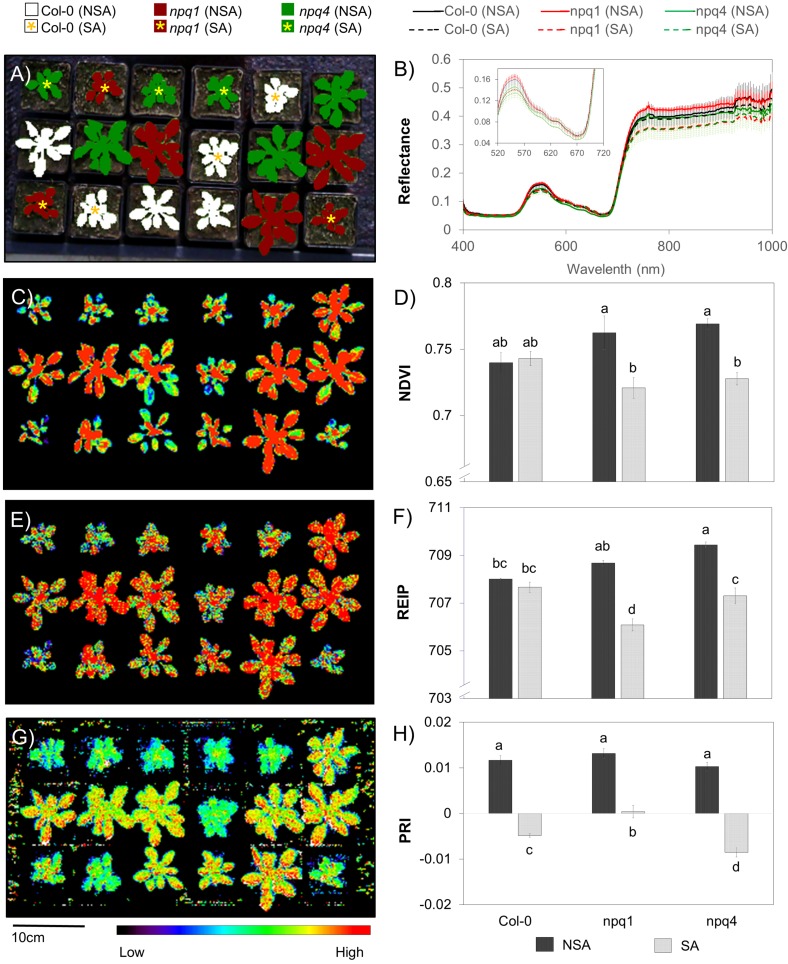
Differences observed between non-stress acclimated (NSA) and stress acclimated (SA) *Arabidopsis* wildtype (Col-0) and NPQ-deficient mutants (*npq1* and *npq4*) as shown by computed spectral ratios. Left panel shows the false-colour images of selected ROIs (**A**); NDVI (**C**); REIP (**E**); and PRI (**G**) computed from spectral information captured by the Specim IQ camera. Right panel shows the computed means ± standard errors of reflectance values (**B**); NDVI (**D**); REIP (**F**); and PRI (**H**) from three individual plants randomly distributed in the imaging frame. Different letters indicate significant differences based on LSD (α=0.05).

Vegetation indices computed from the ROIs of the images of the SA and NSA plants revealed distinct differences in NDVI, REIP and PRI. Plants’ spectral reflectance (Figure 6B) varied in green (∼530 nm), red edge (∼700 nm) and near-infrared (>700 nm) regions of the spectrum where light-use efficiency, narrowband and broadband greenness are estimated, respectively. For both NDVI (Figure 6C,D) and REIP (Figure 6E,F), the two *npq* mutants showed significant changes between SA and NSA while no significant difference was observed in Col-0 plants. Although the shift in red edge inflection point is small (∼2 nm), LSD test showed significant differences between SA and NSA plants of *npq1* and *npq4*. Previous studies [67,68] reported that the red edge shift towards the shorter wavelength was related to a reduced chlorophyll content in leaves. This demonstrates the sensitivity of these indices to subtle changes in pigment ratios and leaf structures which arose in leaves of the NPQ-deficient mutants during acclimation to the stressful condition. The third index, PRI, revealed stress response (Figure 6G) in all plants. The PRI values decreased in all SA plants on average by 1.4-fold and the three genotypes significantly differed (α = 0.01) in the extent of the decrease (Figure 6H). Moreover, fair resolution of pixel patterns within a single plant was captured by the calculated VIs (Figure 6C,E,G) which can further provide spatial information on the relative distribution of different pigments ratios reflecting plant status.

This case study demonstrates the usability of a portable hyperspectral camera to simultaneously detect relative changes in both chlorophyll content (NDVI and REIP) and the xanthophyll cycle (PRI) in Arabidopsis plants. While this case study only showed representative vegetation indices, it offers more opportunity to compute other established VIs (including those which are based on derivatives of spectra) to gain more physiological information in plants. The magnitude of change between the SA and NSA plants can provide quantitative information indicative of stress levels, as shown here in simple rosette of *Arabidopsis thaliana*.

### 4.2. Quantification of Powdery Mildew Infection on Barley

Information on disease severity of crop plants is relevant for the evaluation of the susceptibility of host plants against specific plant pathogens. It is a crucial information source for breeders and essential for plant protection measures in precision agriculture. Hyperspectral imaging, as a non-invasive and objective method, has shown great promise for phenotyping applications in previous studies [5,21,69]. Furthermore, the possibility to create automated measurement series directly in phenotyping greenhouses and fields could lead to an increased throughput for phenotyping applications, reducing the time required for the development of new pathogen resistant cultivars [70]. In the field of precision farming, the early and precise detection of plant diseases is also highly relevant as it determines the efficiency of countermeasures [5].

Therefore, the Specim IQ was tested in a scenario for the assessment of powdery mildew disease severity of different barley cultivars on the canopy scale. The cultivars Milford and Tocada, which have different susceptibility ratings against powdery mildew with 4 and 7 according to the German cultivar list (Descriptive Variety List; Bundessortenamt, Hanover, Germany), were investigated with both camera systems for comparison and evaluated according to the results of subsequent image analysis. The goal of the case study was to accurately detect powdery mildew symptoms on both cultivars. Furthermore, it was of interest to achieve a quantification of the disease symptoms over the image in order to assess the different disease severities of the cultivars through hyperspectral imaging in combination with data analysis methods.

**Figure 7:**
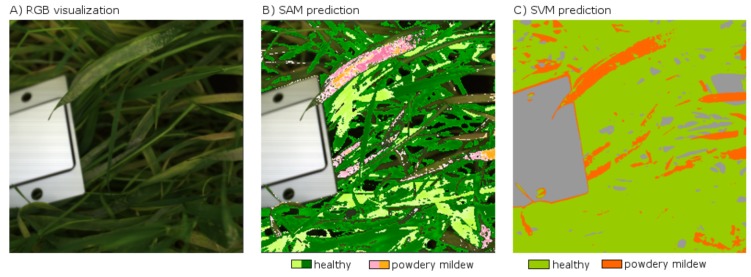
Classification of powdery mildew using the Spectral Angle Mapper (SAM) and Support Vector Machine (SVM). Powdery mildew detection with the SAM is based on two reference spectra for “symptoms” and two reference spectra for “healthy tissue”. The SVM prediction is based on 15 training samples for each class. The image contains the white reference panel on the left side.

For the collection of reflectance characteristics, measurements were conducted using the Specim IQ in a Mini-Plot phenotyping facility in a greenhouse (Thomas et al., in preparation). The “Mini-Plots” are containers which allow the observation of small canopies of up to 360 barley plants with sufficient soil, to enable a root system development similar to barley plants under field conditions. The plants can grow under natural light conditions, while a curtain system at the movable measurement platform allows the application of stable artificial light conditions with diffuse light. The diffuse light is provided by a setup of six 120 W halogen lamps, which are spread over the measurement platform for stable lighting conditions. The properties of the diffuse light lowers the impact of the canopy architecture on the measurement results, due to the reduction of shadowing and different reflection angles in the canopy. Observations were performed on 11/23/2016 at 26 days after inoculation (dai) under controlled light conditions with a measurement distance of around 60 cm and oblique view. The images were normalized using a white reference within the image (cf. Figure 7) and the built-in function of the Specim IQ. A representative result of the SAM analysis of the Specim IQ Studio is given in Figure 7B. Compared to the SVM classification in Figure 7C, the SAM detects similar regions with symptoms in the upper leaves, but misses some of the older, brownish symptoms which are instead not assigned to a class. This may be related to the limited number of samples used for the SAM. Therefore, an increased number of samples may enable competitive results. A quantitative evaluation of the SAM result was not supported by the used version of Specim IQ Studio.

To assess the suitability of the recorded images for the quantification of susceptibility, a SVM classification model for disease symptoms was derived based on manual annotation. “Background”, “healthy leaf tissue” and “leaf tissue with powdery mildew symptoms” were used as target classes. The linear SVM model was learned using 15 annotated samples for each of the three classes. Using the more complex non-linear SVM with a radial function kernel did not improve the result accuracy. The classified background was removed from the further analysis and the ratio of symptomatic pixels to all plant pixels was calculated. For the inoculated (inoc.) cv. Milford and cv. Tocada, disease symptoms were detected at 25.8% and 4.4% of all plant pixels, whereas for the healthy plots (cont.) only 2.0% and 2.2% were determined, respectively. Existing misclassifications were mainly the effect of mixed pixels at the border of the white reference as they appear like the white mycelium on the leaf surface. To remove this systematic bias, the disease severity was determined by subtracting the amount of pixels classified falsely as symptomatic (inoculated-control). Strong difference of 2.2% to 23.7% for the analyzed cultivars was predicted. This values represent the visual impression of the RGB pixels but do not represent the rated susceptibility given by the German cultivar list. The main reason for this is the specific selection of the measured region (approximately 30 × 30 cm), which does not represent the disease severity within the whole plot.

Using the Specim IQ, it was possible to assess the disease severity of complex canopies by measurements in the greenhouse. Controlled illumination conditions support the high signal quality. The spatial resolution was sufficient to identify single symptoms on the barley leaves.

## 5. Discussion

The investigations performed in this study evaluate the Specim IQ camera with regard to spectral imaging quality and usability in typical applications in current plant phenotying research. The comparison of the new Specim IQ camera with the established Specim V10E revealed the competitiveness of the handheld device, but also its limits. In laboratory experiment, high level of conformity was testified on the observed test materials. The visualized spectra in Figure 3 and the mean distances between the spectra of averaged 0.009 (min: 0.004 and max: 0.016) show the overall good measurement quality. At some materials the phenomenon was observed that going from 400 nm to 450 nm the reflectance is decreasing even if the Specim V10E detects a increasing reflectance (cf. Figure 3B). This effect leads to increased distances between the observed spectra if the material has a decreasing reflectance in this region but has nearly no effect if the reflectance is increasing. One reason could be the influence of stray light that adds a systematic effect to first few bands. The option to compensate this effect by calibration or hardware adaption needs further investigations. In the daylight observations, both cameras still show a good coherency, but to a lower degree. Natural variation in the light intensity and composition causes a higher standard deviation of 0.029 compared to 0.019 and an increased mean distance of 0.043 (Figure 4, Table A2). A systematic deviation of the Specim IQ with an absolute value of up to 0.10 was visible in the near infrared from 925 nm to 1000 nm. A line pattern, caused by slight instabilities of the slit width of the Specim IQ sensor resulting in a variability of up to 5% in reflectance, was visible in all images of the Specim IQ (Figure 5). Its effect on the reference measurements was minimized as a region of the image was averaged. However, the Specim IQ showed suitable imaging characteristics in both environments that allow a reliable sensing of reflectance characteristics. The performance under different environmental conditions can be mostly deduced as they will be a mixture of the presented extreme conditions.

The application of the Specim IQ for the investigation of *Arabidopsis thaliana* mutants with different rates of NPQ reveals the suitability of selected VIs and the potential of the Specim IQ to record these subtle parameters (Figure 6). For instance, chlorophyll content was estimated with the use of REIP, which requires a continuous hyperspectral signal as it is based on the first derivative of the vegetation spectra. Using the three indices shown, it was possible to detect a deviation from the wildtype (Col-0) while significant difference was observed between the two mutants under stress condition. In general, hyperspectral imagers are able to provide such comprehensive reflectance information that allow a flexible sensing of various target parameters applicable for high-throughput phenotyping in the lab and in the field. The Specim IQ provides sufficient spectral resolution and radiometric accuracy to record reliably VI values. Thus, a characterization of subtle differences in plant characteristics can be performed by the Specim IQ given a suitable selection of VIs. Hereinafter, it is recommended to investigate the possibility to detect steady-state chlorophyll a fluorescence based on proper derivatives of plant spectra taken under controlled conditions [71].

**Figure 8:**
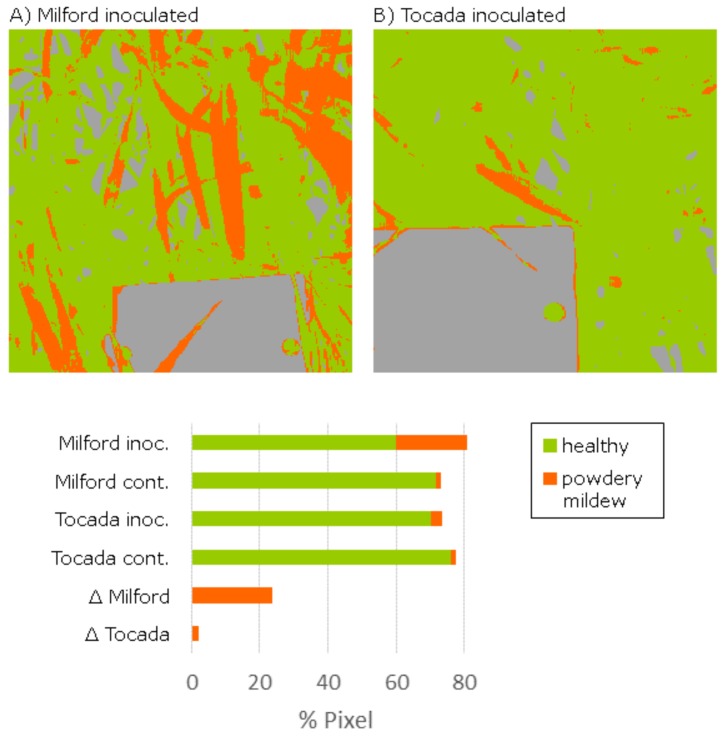
Evaluation for the images of inoculated barley plants by a Support Vector Machine classification model (green: healthy, orange: symptom, gray: background): Cultivar (cv.) Milford shows significantly more affected pixels whereas the cv. Tocada shows only a few symptoms in the measured part of the canopy. Percentage of affected pixels is given for inoculated (inoc.) and healthy control (cont.) plants.

The application of the Specim IQ for the detection and quantification of powdery mildew on barley plants in the Mini-Plot facility showed promising results. The SVM analysis determines reasonable predictions with a good concordance to the visual perception (Figure 8). Applied in larger experiments, the Specim IQ can be used to rank the resistance level of multiple varieties providing significant input in resistance breeding. The quantification of powdery mildew infections based on hyperspectral images is a challenging task due to the likelihood of confusion with specular reflections and senescent leaf parts. Consequently, the SVM classifier was applied, which has been proven to provide convincing results in hyperspectral data sets (Figure 7). A detection of powdery mildew using the SAM, which is integrated in Specim IQ Studio, may be possible by combining multiple reference spectra. Using only few reference spectra, it produces incomplete detection results as not all appearances of the disease were covered. Moreover, a qualitative assessment of the detection accuracy was not possible as an export of classification results was not possible with the software version available during the experiments. The definition and optimization of tailored data interpretation models is decisive for the detection accuracy, whereas the data quality of Specim IQ camera is not a limiting factor.

The handling of the Specim IQ is determined by the predefined imaging pipeline (Figure 2). Within this pipeline, all relevant parameters are requested before the image is taken and operating errors like a missing white reference capture are, as far as possible, prevented. The interface is designed to lower the initial hurdle for the unexperienced operator. The integrated touch screen supports the user by clear instructions compared to the alternative multi-purpose buttons. If suitable analysis programs are deployed on the device, on the fly imaging and analysis can be performed by an inexperienced person after a short introduction. On the other side, such dialogue approach prevents a high level of automation. Furthermore, a remote control from a computer was not supported in the prototype version. Summarizing, in setups with a high level of repetitions, such as static measurement or high-throughput platforms, other sensors are preferable. The Specim IQ sensor is developed for mobile and highly flexible applications in the laboratory, the greenhouse and in the field.

## 6. Conclusions

The new Specim IQ camera was evaluated with regard to the measurement quality, the handling and the performance in real-world scenarios of plant-physiological and agricultural experiments. The radiometric evaluation of the provided Specim IQ prototype revealed a high level of concordance to the established Specim V10E in indoor and outdoor settings. Limitations are revealed for the bands 400–415 nm at some materials and for the bands 925–1000 nm in direct sunlight. Applications are possible indoors and outdoors. The results obtained from the VI analysis suggest potential applications in the context of plant research and phenotyping strategies. For the assessment of powdery mildew, the Specim IQ showed sufficient measurement capabilities and in combination with SVM a high level of consistency to visual assessment in quantification.

The Specim IQ may become a promising hyperspectral camera device with multiple opportunities in plant science. The high measurement quality in combination with compactness, mobility and integrated processing capabilities creates the conditions to acquire new application fields. Trade-off situations between data quality, throughput and stability of environmental conditions are common in plant phenotyping. The Specim IQ allows now to transfer sensor technique at the quality level of laboratory equipment to the greenhouse and the field without any carrier platform or control and storage devices. Thus, the flexible and robust Specim IQ supports the technology transfer to the field and has the potential to increase the overall impact of hyperspectral sensing technologies.

## Figures and Tables

**Table 1:** Specim IQ technical specification.

Parameter	Value
Spectral camera	VNIR 400–1000 nm (CMOS)
Viewfinder camera	5 Mpix
Focus camera	1.3 Mpix
User interface	SW Specim
Processor	NVIDIA Tegra K1
Storage SD card	max. 32 GB
Data format	Specim data set with ENVI compatible data files
Battery	5200 mAh Li-Ion (Type 26650)
Operational time	100 measurements with one SD card and battery
Display & keyboard	4.3” touch screen & 13 physical buttons
Camera interface	USB Type-C
Connectivity	GPS
Size	207×91×74 mm (depth with lens 125.5 mm)
Weight	1.3 kg
F/number	1.7
Wavelength range	400–1000 nm
Spectral resolution FWHM	7 nm
Slit length	42 μm
Slit height	11.7 mm
Spatial sampling	512 px
Spectral bands	204
Image resolution	512×512 px
Data output	12 bit
QE peak	>45%
Full well capacity	>32,000 e-
Peak SNR	>400:1
Working distance	150 mm–*∞*
FOV	$31^{\circ }\times 31^{\circ }$
FOV at 1 m distance	0.55 m × 0.55 m
Temperature, operational	0 °C to +40 °C

## Appendix A

**Table A1:** Comparative performance parameters of the Specim IQ and Specim V10E observed in the laboratory under artificial light conditions. Mean standard deviation of the homogeneous color cards, the mean absolute distance between the mean spectra, the maximum absolute distance and the mean relative distance in percent.

Indoor	Std. Specim V10E [-]	Std. Specim IQ [-]	Mean Abs. Distance [-]	Maximum Abs. Distance [-]	Mean Rel. Distance [%]
Paper, Yellow	0.017	0.022	0.008	0.022	0.019
Paper, Green	0.017	0.023	0.004	0.015	0.006
Paper, Red	0.015	0.016	0.016	0.033	0.098
Paper, bright Yellow	0.018	0.022	0.008	0.031	0.013
Paper, Pink	0.017	0.018	0.006	0.023	0.011
Polyethylene, Pink	0.017	0.025	0.008	0.038	0.013
Polyethylene, Blue	0.017	0.024	0.010	0.030	0.020
Polyethylene, White	0.020	0.032	0.007	0.032	0.008
Polyethylene, Red	0.015	0.017	0.006	0.022	0.033
Polyethylene, Orange	0.016	0.016	0.006	0.037	0.013
Polyethylene, Purple	0.015	0.013	0.004	0.021	0.008
Average	0.016	0.021	0.008	0.028	0.022

**Table A2:** Comparative performance parameters of the Specim IQ and Specim V10E observed outdoors under natural light conditions. Mean standard deviation of the homogeneous color cards, the mean absolute distance between the mean spectra, the maximum absolute distance and the mean relative distance in percent.

Outdoor	Std. Specim V10E [-]	Std. Specim IQ [-]	Mean Abs. Distance [-]	Maximum Abs. Distance [-]	Mean Rel. Distance [%]
Paper, Yellow	0.040	0.046	0.054	0.183	0.072
Paper, Green	0.041	0.047	0.060	0.222	0.077
Paper, Red	0.041	0.035	0.037	0.156	0.090
Paper, bright Yellow	0.031	0.035	0.050	0.193	0.065
Paper, Pink	0.036	0.045	0.051	0.237	0.065
Paper, White	0.040	0.043	0.071	0.254	0.081
Polyethylene, Pink	0.020	0.017	0.024	0.138	0.043
Polyethylene, Blue	0.020	0.016	0.039	0.185	0.057
Polyethylene, White	0.023	0.022	0.023	0.145	0.025
Polyethylene, Red	0.018	0.014	0.041	0.171	0.130
Polyethylene, Orange	0.021	0.018	0.035	0.183	0.084
Polyethylene, Purple	0.018	0.015	0.031	0.160	0.062
Average	0.029	0.029	0.043	0.186	0.071
